# Clinical Features and Outcomes in Adults With Childhood Repair of Partial Atrioventricular Septal Defect

**DOI:** 10.1016/j.jacadv.2022.100007

**Published:** 2022-03-18

**Authors:** C. Charles Jain, William R. Miranda, Heidi M. Connolly, Malini Madhavan, Alexander C. Egbe

**Affiliations:** Department of Cardiovascular Medicine, Mayo Clinic, Rochester, Minnesota, USA

**Keywords:** atrial fibrillation, atrioventricular septal defects, canal, valve regurgitation

## Abstract

**Background:**

Partial atrioventricular septal defects (pAVSDs) are mostly repaired in childhood; however, there are limited data describing these patients in adulthood.

**Objectives:**

The objective of this study was to describe clinical course and associations with outcomes in adults with repaired pAVSDs.

**Methods:**

A retrospective review of adults (≥18 years) with pAVSDs repaired in childhood who presented to the Adult Congenital Heart Disease Clinic at our institution was conducted.

**Results:**

Of 121 patients, the median age was 31 years (IQR: 22-43 years) and 71.9% were female. The median number of operations at the time of presentation was 1 (IQR: 1-2). Left atrioventricular valve (LAVV) replacement had been performed in 19.8% of patients. Among those with native LAVV, 41.2% had ≥ moderate regurgitation. Atrial arrhythmias were present in 34.7% and were associated with later age at repair (*P* = 0.02) and a high number of prior surgeries (*P* = 0.005). Estimated systolic pulmonary artery pressure >40 mmHg was seen in 19.8%. Over 4 (IQR: 1-12) years of follow-up, death occurred in 13 (10.7%) patients and reoperation was required in 39.7%. One-third had a LAVV prosthesis by the end of the study. Atrial fibrillation was independently associated with death or hospitalization on multivariable analysis.

**Conclusions:**

In this cohort of adults with pAVSDs repaired in childhood, atrial fibrillation was common at a young age and associated with worse outcomes. Thus, more studies are needed evaluating the cause of this arrhythmia burden and possible associated atrial myopathy. While many require surgery in adulthood, more information is needed regarding indications for and impacts of LAVV intervention as one-third had an LAVV prosthesis by the end of follow-up.

## Teaser

There are limited data regarding the clinical course of adults with childhood repair of partial atrioventricular septal defects (pAVSDs). In this retrospective cohort of 121 adults, we found a high prevalence of atrial arrhythmias. Atrial fibrillation was found to correlate with death or hospitalization on multivariable analysis. There was no difference in symptoms or outcomes in those with and without significant left atrioventricular valve regurgitation (LAVV). Many required surgery in adulthood, and one-third had an LAVV replacement by the end of follow-up. More studies are needed to better assess and manage atrial arrhythmias and LAVV regurgitation in adults with pAVSDs.

While numerous studies have described the surgical outcomes of patients with partial atrioventricular septal defects (pAVSDs), there is a lack of data regarding their clinical presentation and outcomes in adulthood. Over 60 years ago, Jane Somerville described the natural history in patients with pAVSDs presenting from childhood through adulthood.^1^ This early study described high morbidity and considerable mortality without reparative surgery, given the high prevalence of left atrioventricular valve (LAVV) regurgitation and atrial arrhythmias, along with particularly poor outcomes with pulmonary hypertension as well as endocarditis.^1^

Advances in echocardiography and cardiac surgery have changed the “natural history” over the ensuing years since most patients now undergo repair in childhood, given the feared long-term complications (Central Illustration).^2^^,^^3^ Although there is still some debate on the optimal timing of repair in children, most studies describe excellent early and late outcomes.^2^^,^^4^, ^5^, ^6^ While outcomes of reparative surgery in adults are also favorable,^7^^,^^8^ as more children with repaired pAVSDs survive to adulthood, their clinical features, comorbidities, and outcomes as adults remain unclear.^3^ The purposes of this study were: 1) to describe the clinical presentation and outcomes of adults with repaired pAVSDs; and 2) to evaluate the association between previously described complications following pAVSD repair (eg, atrial fibrillation, LAVV regurgitation) and long-term outcomes.

**Central Illustration:**
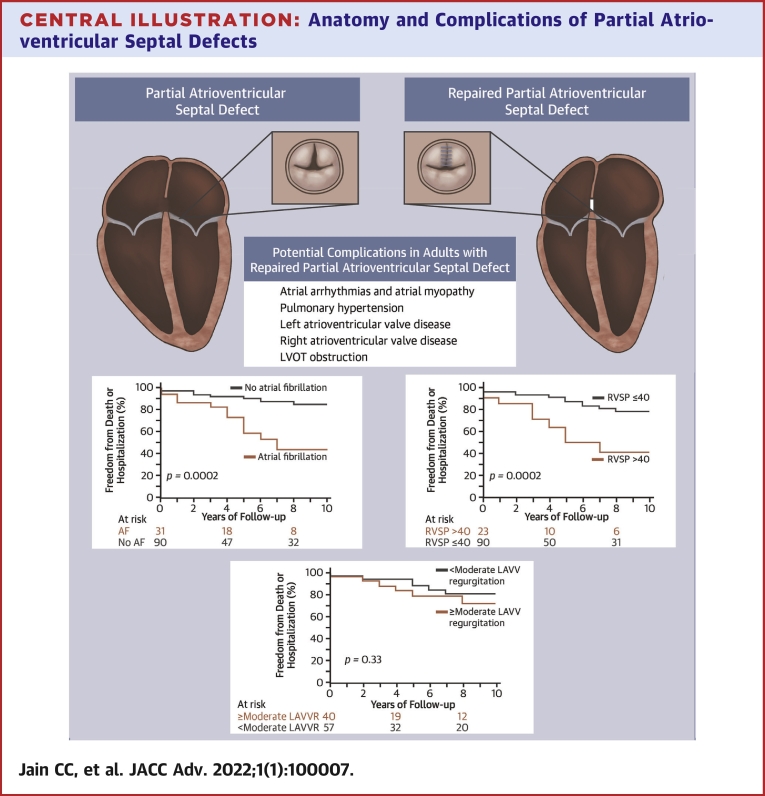
Anatomy and Complications of Partial Atrioventricular Septal Defects **(Top left)** Illustration of unrepaired pAVSDs, showing the ostium primum, both AV valves inserting into the ventricular septum at the same level, and in addition highlighting large atria, an enlarged right heart, and a small left ventricle. The zoomed-in short-axis view of the left AV valve shows the cleft in the anterior leaflet. **(Top right)** Illustration of repaired pAVSDs, showing surgical material in the atrial septum, along with biatrial enlargement and normal sized ventricles. The zoomed-in short-axis view of the left AV valve shows the repaired cleft. AV = atrioventricular; pAVSD = partial atrioventricular septal defect.

## Methods

### Study population

A total of 121 adults (≥18 years of age) with pAVSDs that underwent repair in childhood or adolescence (<18 years of age) and were seen between January 2000 and December 2018 were retrospectively identified through the Mayo Adult Congenital Heart Disease registry. Anatomy was confirmed by operative report details when available (n = 81) and by echocardiography and clinical notes for the remainder (n = 40). The anatomy of pAVSDs was defined as an atrial level shunt in the typical ostium primum location in the setting of 2 distinct atrioventricular valves.^9^ Seven patients (5.8%) were felt to have a transitional atrioventricular septal defect, either with a small ventricular septal defect or with a small aneurysm in the inlet ventricular septum suggestive of prior ventricular septal defect. All patients provided authorization for the use of their medical records in research, and the Institutional Review Board approved the study.

### Data collection

The medical records including clinical, echocardiographic, laboratory, and surgical data were reviewed. Baseline data reflect information available at the patients' first cardiology visit as an adult (≥18 years) at our institution. Two-dimensional echocardiography and Doppler echocardiography were performed for all patients. The severity of valvular regurgitation was assessed using qualitative Doppler echocardiography and reflects the echocardiographer's assessment at the time of clinical evaluation. Patients with an LAVV prosthesis at the time of echocardiogram were excluded from analysis of LAVV inflow and tissue Doppler echocardiography. Doppler echocardiography–estimated right ventricular systolic pressure (RVSP) >40 mmHg was used to screen for pulmonary hypertension and was estimated as the peak right atrioventricular valve velocity plus estimated right atrial pressure, in accordance with guidelines.^10^^,^^11^ In those with right ventricular outflow obstruction, the maximal instantaneous gradient across the right ventricular outflow tract was subtracted from the RVSP to avoid overestimation of the pulmonary artery systolic pressure.^12^

The following outcomes were assessed: 1) heart failure-related and arrhythmia-related hospitalization; 2) all-cause mortality; 3) combined death or heart failure–related or arrhythmia-related hospitalization. Mortality was ascertained from the medical records and the Accurint database, an institutionally approved location service.^13^ Heart failure–related hospitalization was defined as admission for volume overload requiring intravenous diuretics, while arrhythmia-related hospitalization was defined as nonelective admission for symptomatic, uncontrolled arrhythmias (thus excluding elective hospitalizations for procedures or medication initiation).

### Statistical analysis

Continuous variables are presented as mean ± SD for nominal variables and median (IQR) for nonparametric distributions. Categorical variables were presented as counts (%). The primary outcome was a combined end point of death or hospitalization for heart failure or symptomatic, uncontrolled arrhythmia. Between-group comparisons were performed using unpaired t-tests, analysis of variance, Wilcoxon signed rank test, Fisher exact test, and Pearson's correlation coefficient. Simple linear regression was used to assess associations between continuous variables. The Kaplan-Meier analysis was used for univariable assessment of outcomes with Wilcoxon signed rank test. A multivariable Cox model for outcomes was performed using age, left ventricular ejection fraction, and New York Heart Association (NYHA) class in a forward selection. The strength of association for each variable was expressed using hazard ratio (HR) and 95% confidence intervals (CIs). All statistics were performed with JMP software (version 14.1.0, SAS Institute Inc.). A value of *P* < 0.05 was considered statistically significant.

## Results

### Baseline characteristics

Of 121 adults with pAVSDs repaired in childhood, the median age was 31 (IQR: 22-43) years and 71.9% were female (Table 1). The median age of repair was 4 (IQR: 2-8) years. Genetic syndromes were diagnosed in 13 patients (10.7%), with Down syndrome being the most common one (n = 9) followed by CHARGE syndrome (n = 2), Noonan syndrome (n = 1), and Bardet-Biedl syndrome (n = 1). Associated defects were present in 29 (23.9%) individuals, with pulmonary stenosis being the most common one (n = 7, 5.8%), followed by other atrioventricular valve abnormalities (eg, parachute LAVV) (n = 4, 3.3%), secundum atrial septal defects (ASDs) (n = 4, 3.3%), small noninlet ventricular septal defects (n = 3, 2.5%), coarctation of the aorta (n = 2, 1.7%), and other (eg, coronary anomalies, cardiac malposition or situs inversus) (n = 8, 6.6%).

**Table 1:** Patient Characteristics

Variable	N	All
Demographics		
Age (y)	121	31 (22-43)
Female (%)	121	87 (71.9)
Body mass index (kg/m^2^)	121	25.1 (22.3-31.0)
Associated genetic syndromes (%)	121	13 (10.7)
Age at repair (years)	121	4 (2-8)
Anatomy details	121	
Transitional (%)	121	7 (5.8)
Associated defects (%)	121	29 (23.9)
Small VSD (noninlet)	121	3 (2.5)
Patent ductus arteriosus	121	2 (1.7)
Secundum atrial septal defect	121	4 (3.3)
Noncleft AV valve abnormalities	121	4 (3.3)
Coarctation of the aorta	121	2 (1.7)
Pulmonic stenosis	121	7 (5.8)
Other	121	8 (6.6)
Surgical history		
Left AV valve repair at initial surgery (%)	81	70 (86.4)
Right AV valve repair at initial surgery (%)	81	1 (1.2)
Subaortic stenosis resection at initial surgery (%)	81	5 (6.2)
# Operations in childhood (age <18 years)	121	1 (1-2)
# Operations at the time of presentation	121	1 (1-2)
Symptoms and comorbidities		
NYHA functional class III-IV (%)	121	25 (20.7)
Atrial arrhythmias (%)	121	42 (34.7)
Atrial fibrillation (%)	121	31 (25.6)
Atrial flutter (%)	121	12 (9.9)
Ventricular arrhythmias (%)	121	5 (4.1)
Pacemaker (%)	121	22 (18.2)
Hypertension (%)	121	16 (13.2)
Hyperlipidemia (%)	121	8 (6.6)
Diabetes mellitus (%)	121	6 (5.0)
Smoking, current or prior (%)	121	16 (13.2)
Obstructive sleep apnea (%)	121	12 (9.9)
Medications		
Beta blocker (%)	121	26 (21.5)
Calcium channel blocker (%)	121	6 (5.0)
ACEi/ARB (%)	121	29 (24.0)
Aldosterone antagonists (%)	121	8 (6.6)
Antiarrhythmics (%)	121	9 (7.4)
Pulmonary vasodilators (%)	121	0 (0.0)

Values are n, median (IQR), or n (%).

ACEi/ARB = angiotensin-converting enzyme inhibitor/angiotensin receptor blocker; AV = atrioventricular; NYHA = New York Heart Association; VSD = ventricular septal defect.

The vast majority (86.4%) underwent LAVV repair at the time of initial operation surgical repair, while 6.2% underwent relief of subaortic stenosis and only 1.2% underwent right atrioventricular valve repair at the time of initial surgical repair. The median number of surgeries at the time of presentation was 1 (1, 2). An LAVV prosthesis was present in 24 patients (19.7%).

**Figure 1:**
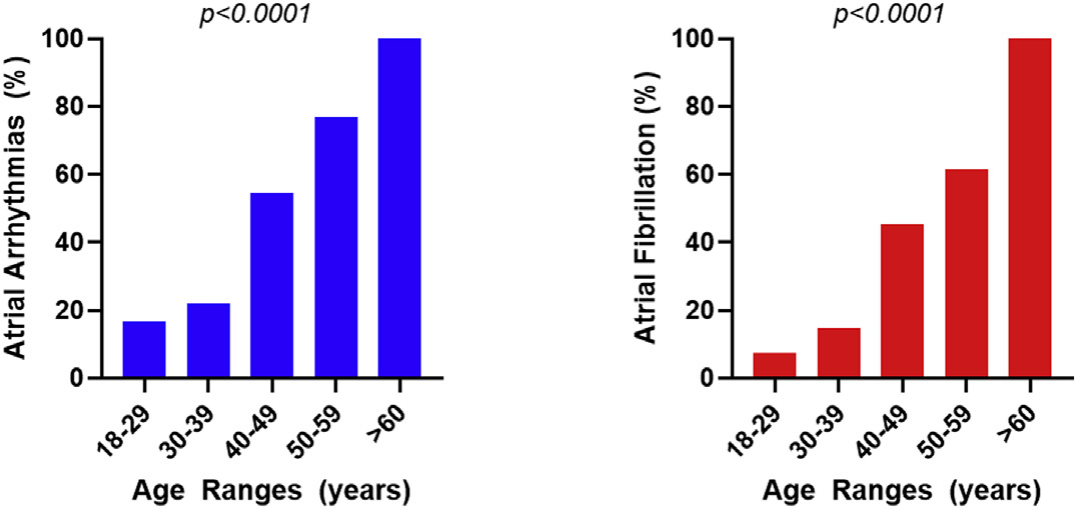
Frequency of Atrial Arrhythmias and Atrial Fibrillation in Adults With Partial Atrioventricular Septal Defects These histograms show the percentage of patients with atrial arrhythmias **(left)** and atrial fibrillation in particular **(right)** by age. While atrial arrhythmias are found in many patients <40 years of age, once ≥40 years of age, the majority have atrial arrhythmias, predominantly atrial fibrillation.

At the time of presentation, 25 patients (20.7%) had NYHA functional class III-IV. Over one-third of patients (34.7%) had prior atrial arrhythmia, with a history of atrial fibrillation present in 25.6% (paroxysmal in 23 [19.0%] and persistent in 8 [6.6%]). The prevalence of atrial arrhythmia increased significantly with age (*P* < 0.0001) from 16.7% of those <30 years (n = 54) to 83.3% of those ≥50 years (n = 18) with comparable findings for the prevalence of atrial fibrillation (7.4% vs 72.2%; *P* < 0.0001) (Figure 1). Those with atrial fibrillation were older at the initial repair (5 [IQR: 4-10] vs 4 [IQR: 1-6]; *P* = 0.02) and had a higher total number of operations (3 [IQR: 2-4] vs 2 [IQR: 1-3]; *P* = 0.005). Pacemakers had been implanted in 22 (18.2%) patients.

Laboratory, echocardiography, and exercise testing data are described in Table 2. At the time of echocardiography, 90.0% were in sinus rhythm (90.0%), 8.3% in atrial fibrillation, and 1.7% in atrial flutter. The left ventricular ejection fraction was 59 ± 9%. Notably, the left ventricular linear dimensions were normal despite 41.2% having ≥ moderate native LAVV regurgitation. The mean gradient through the LAVV was 4 (3, 6) mmHg. The medial E/e' was 14 (10, 19). Right atrioventricular valve regurgitation ≥ moderate was present in 22.3%, and 16.5% had ≥ moderate right ventricular enlargement. The RVSP was 34 ± 12, >40 mmHg in 19.8% patients and >60 mmHg in 6.2% patients. No patients were on pulmonary vasodilators. The RVSP had a significant correlation with left atrial volume index (r = 0.55; *P* < 0.0001), and those with enlarged left atrial volume index (>34 ml/m^2^) were more likely to have a higher RVSP (38 ± 16 vs 30 ± 8 mmHg; *P* = 0.0008).

**Table 2:** Laboratories, Echocardiography, and Exercise Testing

Variable	N	All
Laboratories		
Creatinine (mg/dL)	112	0.9 (0.7-1.0)
NT-proBNP (pg/mL)	41	329 (161-1271)
Echocardiography		
LV ejection fraction (%)	121	59 ± 9
LV EDD (mm)	121	50 ± 7
LV ESD (mm)	121	32 ± 7
Left atrial volume index (ml/m^2^)	99	38 (28-57)
Left AV valve prosthesis (%)	121	24 (19.8)
Right AV valve prosthesis (%)	121	0 (0.0)
Aortic valve prosthesis (%)	121	3 (2.5)
Pulmonic valve prosthesis (%)	121	1 (0.8)
≥Moderate native left AV valve regurgitation (%)^a^	97	40 (41.2)
Mean gradient native left AV valve (mmHg)^a^	48	4 (3-6)
Left AV valve area (cm^2^)^a^^,^^b^	44	2.2 (1.6-2.7)
E velocity (m/s)^a^	81	1.3 ± 0.4
A velocity (m/s)^a^	68	0.8 ± 0.3
E/A velocity^a^	68	1.6 (1.3-2.4)
Medial E/e'^a^	47	14 (10-19)
Lateral E/e'^a^	38	8 (7-11)
≥Moderate native aortic regurgitation (%)	119	4 (3.4)
Peak native LVOT velocity (m/s)	102	1.6 (1.4-2.2)
≥Moderate RV enlargement (%)	121	20 (16.5)
≥Moderate RV dysfunction (%)	121	12 (9.9)
RVSP (mmHg)^c^	107	34 ± 12
Pulmonary hypertension (RVSP >40) (%)^c^	107	21 (19.8)
Right atrial pressure (mmHg)	120	7 ± 3
≥Moderate native right AV valve regurgitation (%)	121	27 (22.3)
Mean native gradient right AV valve (mmHg)	2	2.5 (2-3)
Exercise testing		
Peak heart rate (bpm)	52	155 (116-173)
% Peak heart rate	52	82 (65-92)
Peak VO_2_ (ml/kg/min)	39	21.8 ± 7.4
% Predicted peak VO_2_	38	63.0 ± 22.0
METS	43	6.4 ± 2.4
% Predicted functional aerobic capacity	41	67.3 ± 19.3
V_E_/V_CO2_	37	28.9 ± 4.7
Peak oxygen saturation (%)	39	97 ± 3
Breathing reserve (%)	36	42.8 ± 18.8

Values are n, median (IQR), mean ± SD, or n (%).

AV = atrioventricular; EDD = end-diastolic diameter; ESD = end-systolic diameter; LVOT = left ventricular outflow tract; METS = metabolic equivalents; RVSP = right ventricular systolic pressure; V_E_/V_CO2_ = ventilatory efficiency; VO_2_ = oxygen consumption.

a Excludes patients with left atrioventricular valve prosthesis.

b Valve area calculated by continuity equation.

c Excludes patients with a history of pulmonic stenosis.

The prevalence of atrial arrhythmias (30.0% vs 26.3%; *P* = 0.82) and atrial fibrillation (22.5% vs 15.8%; *P* = 0.44) was similar between those with and without ≥ moderate LAVV regurgitation (Supplemental Table 1). Those with a history of atrial arrhythmias had significantly larger left atria (64 [IQR: 49-86] vs 33 [IQR: 25-42] ml/m^2^; *P* < 0.0001). While indexed left ventricular dimensions and ejection fractions were comparable between those with and without ≥ moderate LAVV regurgitation, Doppler echocardiography–derived stroke volume index tended to be smaller in those with ≥ moderate LAVV regurgitation (47 ± 13 vs 53 ± 15 ml/m^2^; *P* = 0.09).

### Follow-up

**Figure 2:**
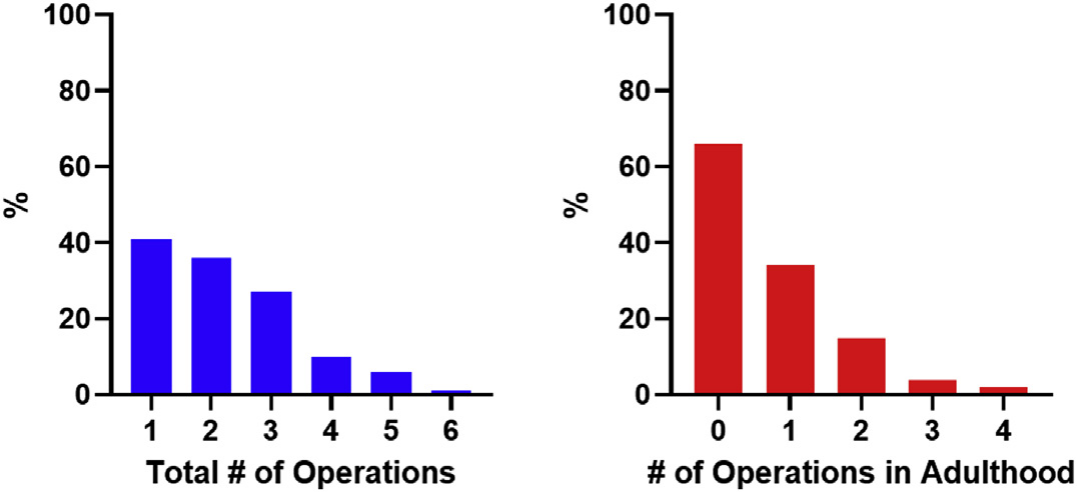
Frequency of Operations in Adults with Partial Atrioventricular Septal Defects The **bar graph on the left** shows the total number of operations that patients underwent from birth through the end of the study period. This highlights that approximately two-thirds warrant reintervention and over a quarter need ≥3 or more surgeries.

Median follow-up was 4 (IQR: 1-12) years, and the information regarding clinical events during follow-up is summarized in Table 3. Reoperation during follow-up occurred in 39.7% of patients (Figure 2). During adulthood, 28.1% underwent 1 operation, 12.4% underwent 2 operations, 3.3% underwent 3 operations, and 1.7% underwent 4 operations. Native LAVV dysfunction was the most common indication for reoperation, and there was no difference in the proportion of patients who underwent subsequent LAVV surgery between those with ≥ moderate LAVV regurgitation and those with < moderate LAVV regurgitation at baseline (35.0% vs 29.8%; *P* = 0.66).

**Table 3:** Outcomes

	N	All
Follow-up (y)	121	4 (1-12)
Surgical outcomes		
# Total operations	121	2 (1-3)
# Operations in adulthood	121	0 (0-1)
# Operations during follow-up	121	0 (0-1)
Operations post-initial repair (%)		
Left AV valve repair	121	34 (28.0)
Left AV valve replacement	121	41 (33.9)
Any left AV valve intervention	121	61 (50.4)
Right AV valve repair	121	17 (14.0)
Right AV valve replacement	121	9 (7.4)
Any right AV valve intervention	121	25 (20.7)
Any surgery for subaortic stenosis	121	21 (17.4)
Interval from left AV valve to subaortic surgery (years)	21	4 (2-9)
Aortic valve replacement	121	4 (3.3)
Redo intervention for atrial septal defect	121	13 (10.7)
AV valve operations during follow-up (%)		
Left AV valve repair	121	15 (12.4)
Left AV valve replacement	121	21 (17.4)
Right AV valve repair	121	13 (10.7)
Right AV valve replacement	121	9 (7.4)
Nonsurgical outcomes		
Heart failure hospitalization (%)	121	12 (9.9)
Arrhythmia hospitalization (%)	121	11 (9.1)
Transplant (%)	121	0 (0.0)
Death (%)	121	13 (10.7)
Death or hospitalization for heart failure or arrhythmia (%)	121	32 (26.4)
Age at death (years)	13	53 (40-62)

Values are n or median (IQR).

AV = atrioventricular.

A total of 13 patients (10.7%) died during follow-up, with a median age of death of 53 (IQR: 40-62) years. Notably only one of the patients who died had a known genetic syndrome. All deceased patients had a history of atrial arrhythmia (12 with atrial fibrillation, 1 with atrial flutter), and the RVSP was higher in those who died (49 ± 14 vs 34 ± 13 mmHg, *P* = 0.0003).

A combination of death or hospitalization for heart failure and/or arrhythmia was documented in 26.4% of patients. Heart failure–related hospitalization tended to occur more often in patients with atrial fibrillation (19.4% vs 6.7%; *P* = 0.07), but this difference was not seen in those with atrial flutter (8.3% vs 10.1%; *P* = 0.99). Twelve patients (9.9%) underwent percutaneous ablation for atrial arrhythmia during follow-up, including 4 patients (3.3%) who did not have atrial arrhythmias at baseline. Endocarditis was seen in 4 patients (3.3%) during follow-up.

**Figure 3:**
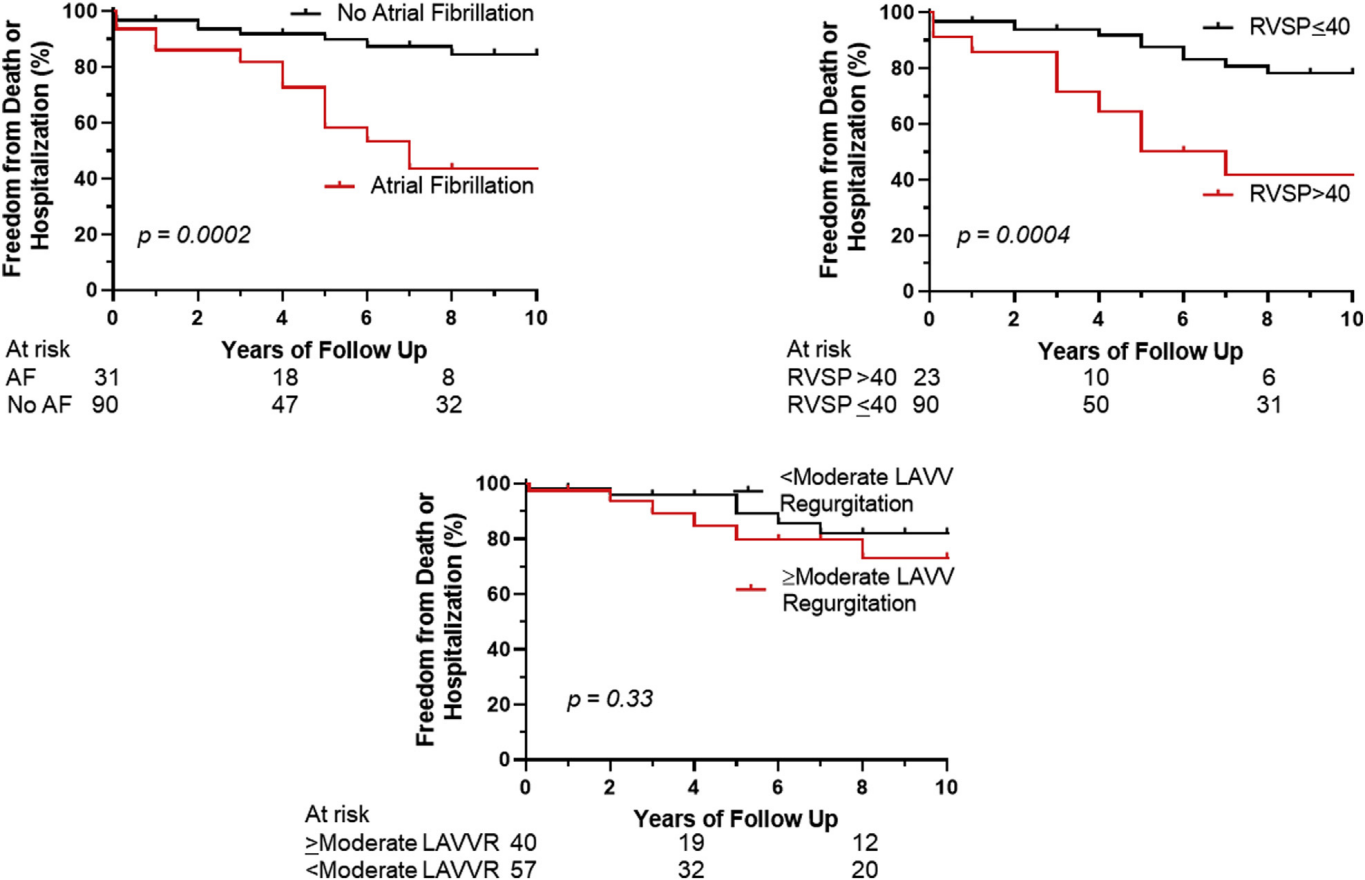
Risk Factors for Death or Cardiovascular Hospitalization in Adults With Partial Atrioventricular Septal Defects On Kaplan-Meier analysis, there was a clear association between outcomes and atrial fibrillation (AF) as well as pulmonary hypertension (as indicated by corrected right ventricular systolic pressure [RVSP]), but no association seen with ≥moderate left atrioventricular valve (LAVV) regurgitation (LAVVR).

Kaplan-Meier survival analysis showed that death was significantly more likely in those with atrial fibrillation (4-year survival rate: 79.3% vs 98.3%; *P* < 0.0001) and pulmonary hypertension (68.9% vs 98.1%; *P* < 0.0001), but no difference was seen in those with NYHA class III-IV (89.3% vs 93.6%; *P* = 0.82) or those with ≥ moderate native LAVV regurgitation (84.8% vs 96.8%; *P* = 0.67) (Supplemental Figure 1). Kaplan-Meier survival analysis showed a composite of death or hospitalization for heart failure or arrhythmia was more likely for those with atrial fibrillation (freedom from death or hospitalization at 4 years was 86.3% vs 91.9% in those with and without atrial fibrillation, respectively; *P* = 0.0002), pulmonary hypertension (*P* = 0.0004), and NYHA class III-IV (*P* = 0.02) (Figure 3). In contrast, the incidence of death or hospitalization for heart failure or arrhythmia was not associated with ≥moderate LAVV regurgitation (*P* = 0.33) or age at initial repair (*P* = 0.83).

When controlling for age, left ventricular ejection fraction, and NYHA class, atrial fibrillation (HR: 3.9, 95% CI: 1.69-9.11, *P* = 0.001) was significantly associated with death or hospitalization for heart failure or arrhythmia during follow-up. This association was also found when assessing for pulmonary hypertension with RVSP >40 mmHg (HR: 3.1, 95% CI: 1.36-7.10, *P* = 0.007), which was no longer present when controlling for atrial fibrillation (*P* = 0.12). There was no significant association between death or hospitalization with ≥moderate LAVV regurgitation (HR: 1.48, 95% CI: 0.51-4.31, *P* = 0.47). Similarly, there was no association between atrial flutter and death or hospitalization (HR: 1.29, 95% CI: 0.42-3.96, *P* = 0.65).

## Discussion

We describe herein the clinical course of patients ≥18 years presenting to the Adult Congenital Heart Disease Clinic who had a history of pAVSDs repaired in childhood. The main findings of our study are as follows: 1) atrial arrhythmias were present in over a third of this relatively young cohort; 2) approximately one-fifth of patients had pulmonary hypertension; 3) significant LAVV regurgitation was present in more than a third of patients, and one-third had an LAVV prosthesis by the end of follow-up; 4) over a median 4 years of follow-up, 10.7% of patients died and 26.4% were hospitalized or died; 5) atrial fibrillation was independently associated with outcomes. Despite the favorable surgical outcomes in the pediatric population, the findings of this study suggest that pAVSDs are associated with significant morbidity in adulthood.

In Paul Wood's early clinical review of patients with ostium secundum ASDs, he emphasized the importance of the loading to the right heart and the feared consequences of pulmonary hypertension as well as atrial arrhythmias.^14^ He described that for many patients, symptoms would not occur until the onset of atrial arrhythmias, which often did not present until >30 years of age. Subsequently Jane Somerville looked specifically at unrepaired pAVSDs and found that atrial arrhythmias presented at even younger ages. She hypothesized that the high prevalence of LAVV disease caused additional load to the atria, and this idea is similar to the atrial fibrosis and remodeling which has subsequently been shown at the gross and molecular level in chronic acquired LAVV disease.^15^ Left atrial dilation and remodeling has been shown to correlate with atrial arrhythmias in patients with secundum defects,^16^ and the concomitant LAVV disease in pAVSDs likely contributes to further left atrial remodeling. The associations of the left atrial size with RVSP and atrial arrhythmias, even when excluding those with significant LAVV disease or left ventricular dysfunction, also serve as evidence of a left atrial myopathy by suggesting elevated left atrial pressure and therefore noncompliance out of proportion to LAVV or left ventricular disease.

### Burden of atrial arrhythmias

Despite the young age at pAVSD repair in our cohort, there was still a high prevalence of atrial arrhythmias, and the prevalence increased significantly with age. While repair may delay the onset of atrial arrhythmias compared to historical data of unrepaired pAVSDs, it may not decrease their lifetime risk. These findings are consistent with data in adults with secundum defects in that age at repair is associated with atrial fibrillation.^17^ Notably, the prevalence of atrial fibrillation in our cohort is even higher than in some other congenital heart diseases in adults such as tetralogy of Fallot or Shone's complex.^18^, ^19^, ^20^ Despite this, only a few patients were treated with antiarrhythmic medications. This high burden of atrial arrhythmias suggests that there is significant remodeling of the left atrium, but whether this is intrinsic and/or due to surgery in childhood remains unclear. As all of the patients who died in this cohort had atrial arrhythmias and atrial fibrillation was significantly associated with poor outcomes on multivariable analysis, these arrhythmias appear not to be well tolerated in adults with repaired pAVSDs.^21^^,^^22^ This combination of intrinsic LAVV disease along with frequent atrial arrhythmias and prior surgery involving the atria likely reciprocates itself and further contributes to a potential atrial myopathy in patients with pAVSDs.

### Pulmonary hypertension in pAVSDs

Interestingly, Somerville also found a lower incidence of pulmonary hypertension in patients with pAVSDs than that Wood found in secundum ASDs. While one-fifth of current cohort had elevations in pulmonary pressures, none were treated with pulmonary vasodilators, which might have been related to the mild degree of pulmonary hypertension in most patients or due to concern for post-capillary/left heart disease in patients with universal abnormalities of the LAVV.^23^^,^^24^ The reasoning behind why patients with pAVSDs, repaired or not, are less likely to develop pre-capillary pulmonary hypertension has yet to be elucidated. Further characterization of the contributions to pulmonary hypertension in pAVSDs is needed.

### Assessment and management of LAVV regurgitation

The burden of LAVV disease in pAVSDs is well described as the most common cause for reoperation.^25^ Interestingly in this cohort, patients with ≥ moderate LAVV regurgitation had a comparable burden of symptoms, atrial arrhythmias, and outcomes as those without significant LAVV regurgitation. While the degree of LAVV regurgitation could be questioned, the accuracy is supported by the lower Doppler echocardiography–derived stroke volume in those with ≥ moderate LAVV regurgitation, consistent with more regurgitation. The comparable left ventricular dimensions between groups could be due to how left ventricles respond to underfilling prior to atrial septal defect repair with abnormal compliance even post-repair.^26^^,^^27^ Alternatively, it is possible that the inherent LAVV stenosis, albeit mild in most, is enough to protect the left ventricle from significant volume loading. However, data from acquired heart disease suggest less than severe LAVV regurgitation can still cause left ventricular remodeling and symptoms and thus warrant surgery.^28^^,^^29^ Further work is needed to better define the criteria (including quantitative echo-Doppler data) for intervention in patients with pAVSDs with asymptomatic as well as symptomatic LAVV regurgitation.

Current guidelines for acquired valve disease as well as congenital heart disease would consider ejection fraction <60% and atrial fibrillation as potential indications for valve surgery in patients with significant LAVV regurgitation.^30^, ^31^, ^32^, ^33^ If these guidelines were followed, then many patients in this cohort would warrant surgery. Compared to acquired mitral valve pathology, patients with pAVSDs have longer duration of ≥ moderate LAVV regurgitation and prior atrial and ventricular insults from the initial unrepaired lesion and subsequent early surgery. Thus, it is likely that the guideline indications for valve surgery for acquired mitral valve disease do not fully apply to adults with pAVSDs. In addition, when considering surgery for the LAVV (particularly elective), the likelihood of valve repairability has significant implications. As the majority had prior LAVV repair, some of these valves may not be amenable to re-repair and notably one-third of this young cohort had an LAVV prosthesis at the end of follow-up.^34^ Thus, perhaps the approach to LAVV surgery in pAVSDs should not be the same as that directed by the guidelines for LAVV disease overall. More data are needed in adults with pAVSD to determine a standardized approach for management of LAVV regurgitation and whether LAVV intervention (particularly replacement) can improve outcomes.

### Outcomes

While only 20.5% of adults in this cohort had significant symptoms at presentation, over follow-up, 26.2% were either hospitalized for cardiac symptoms and/or died. Clinicians should be aware how this is in stark contrast to the excellent long-term outcomes of patients with secundum ASDs repaired in childhood and perhaps pAVSDs should not be considered a “simple” congenital heart disease.^35^ Similar to those with secundum defects, later repair was found to correlate with atrial arrhythmias and poor outcomes. We hypothesize that the longer duration of an unrepaired lesion along with the chronic LAVV disease post-repair are the substrate for high incidence of atrial arrhythmias and this along with any atrial or left ventricular myopathy contribute to the poor outcomes. As mentioned earlier, all patients who died had atrial arrhythmias and atrial fibrillation was independently associated with outcomes on multivariable analysis. While intuitively pulmonary hypertension and ≥ moderate LAVV regurgitation are considered harbingers of poor outcomes in pAVSDs, we found no significant association between these variables and outcomes on multivariable analysis (if controlling for atrial fibrillation). Further investigation is needed to better identify adults with repaired pAVSDs who are at high risk for complications and also how to improve long-term outcomes in these patients.

### Study Limitations

This study is limited by its design as it is a retrospective review of a single-center experience, and as a tertiary care center, the incidence of surgical events may reflect potential referral bias. However, currently there are limited data regarding the clinical course of adults with pAVSDs repaired in childhood. Duration of follow-up is relatively short and thus may underestimate the prevalence of reoperation or clinical events. Assessment for left atrial myopathy was limited by lack of strain and invasive hemodynamic data. Transthoracic echocardiography was used to assess for pulmonary hypertension. While the gold standard for diagnosis of pulmonary hypertension is cardiac catheterization, this could not be used, given the retrospective nature of the study and current clinical practice where catheterization is only performed in a minority of patients. Given the age of patients included in the cohort and reflecting the clinical and surgical practice at the time, the median age of repair is older than that of current cohorts. Given the remote nature of the childhood surgery and our referral practice, operative reports were not available for all patients who underwent surgery at other centers. Lastly, for unclear reasons, there was a higher prevalence of females to males in our cohort, which may potentially limit the generalizability of our findings.

## Conclusions

In this cohort of adults with pAVSDs repaired in childhood, we found a high prevalence of LAVV regurgitation, pulmonary hypertension, and atrial arrhythmias. Atrial fibrillation was associated with worse outcomes and raises the question of a potential atrial myopathy in these patients. Notably, atrial fibrillation was associated with later age at repair and this may have implications for pediatric management. Reoperation was common in adulthood. This study emphasized that adults with pAVSDs repaired in childhood are not “cured” of their structural heart disease,^36^ and we hope this study will guide clinicians in the clinical care of these patients. Further studies are needed to better understand LAVV regurgitation as well as indications and benefits of intervention. In addition, further investigation is needed regarding a potential left atrial myopathy along with characteristics and optimal management of atrial fibrillation.

Perspectives **COMPETENCY IN MEDICAL KNOWLEDGE:** There are limited data describing the clinical course of adults with pAVSDs repaired in childhood. While traditionally pAVSDs are considered to be a “simple” form of congenital heart disease, they have significant prevalence of atrial arrhythmias in early adulthood and this raises the question of a left atrial myopathy. LAVV regurgitation is common, and repair is not possible in all patients as one-third underwent LAVV replacement.

**TRANSLATIONAL OUTLOOK:** Future studies should explore the mechanisms behind the atrial arrhythmia burden and best treatment options. In addition, more information is needed for better quantification of LAVV regurgitation along with studies looking at the impacts of LAVV surgery, particularly replacement.

## Funding support and author disclosures

The MACHD Registry is supported by the Al-Bahar Research grant. Dr Egbe is supported by National Heart, Lung, and Blood Institute (NHLBI) grant K23 HL141448. All other authors have reported that they have no relationships relevant to the contents of this paper to disclose.
