# Supplementary material for: Clinical Features and Outcomes in Adults With Childhood Repair of Partial Atrioventricular Septal Defect
Source: JACC Adv. 2022 Mar 18;1(1):100007. doi: 10.1016/j.jacadv.2022.100007 (PMC11198577; doi:10.1016/j.jacadv.2022.100007)
Supplement: Supplementary Table and Figure [file mmc1.pdf]

Supplemental Table 1. Impact of LAVV regurgitation

	<Moderate native LAVV regurgitation (n=57)	≥Moderate native LAVV regurgitation (n=40)	p value
Age (years)	27 (21, 37)	38 (27, 48)	0.002
Female (%)	44 (77.2)	28 (70.0)	0.48
BMI (kg/m^2)	25.1 (22.5, 28.7)	27.1 (22.3, 33.7)	0.18
NYHA ≥III (%)	10 (17.5%)	8 (20.0%)	0.80
Atrial arrhythmia (%)	15 (26.3%)	12 (30.0%)	0.82
Atrial fibrillation (%)	9 (15.8%)	9 (22.5%)	0.44
LV EDD (mm)	48±6	52±7	0.02
LV ESD (mm)	31±6	34±8	0.10
LV EDD / BSA (m^2)	27.6±4.2	27.5±4.7	0.94
LV ESD / BSA (m^2)	17.8±3.6	17.7±4.3	0.84
LV EF (%)	59±8	59±11	0.76
LV stroke volume index (ml/m^2)	53±15	47±13	0.09
LAVI (ml/m^2)	33 (23, 44)	41 (30, 53)	0.04
E (m/s)	1.3±0.4	1.4±0.4	0.29
E/A	1.6 (1.3, 2.3)	1.6 (1.4, 2.4)	0.65
Medial E/e’	15 (10, 19)	13 (10, 19)	0.65
Lateral E/e’	10 (7, 14)	8 (6, 10)	0.10
RVSP (mmHg)	30 (25, 34)	32 (28, 44)	0.05
≥Moderate RV enlargement	11 (19.3%)	6 (15.0%)	0.79
≥Moderate RV dysfunction	5 (8.8%)	5 (12.5%)	0.74
≥Moderate RAVV regurgitation	9 (15.8%)	12 (30.0%)	0.13

Abbreviations: BMI = body mass index; BSA = body surface area; EDD = end-diastolic diameter; EF = ejection fraction; ESD = end-systolic diameter; LAVI = left atrial volume index; LAVV = left atrioventricular valve; LV = left ventricle; NYHA = New York Heart Association; RAVV = right atrioventricular valve; RVSP = right ventricular systolic pressure

Supplemental Figure 1. Risk Factors for Death in Adults with Partial Atrioventricular Septal Defects

On Kaplan-Meier analysis for death alone, there was a clear association between outcomes and atrial fibrillation (AF) as well as pulmonary hypertension (as indicated by corrected right ventricular systolic pressure, RVSP), but no association seen with $\geq$moderate left atrioventricular valve (LAVV) regurgitation.

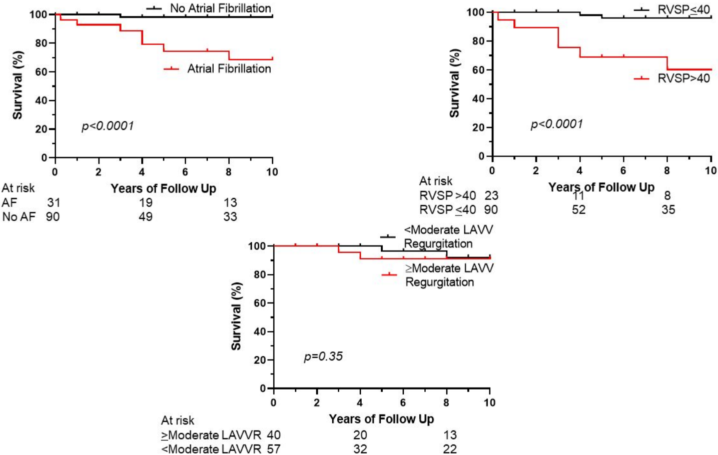
No Atrial Fibrillation
Atrial Fibrillation
Survival (%)
p<0.0001
Years of Follow Up
At risk
AF 31 19 13
No AF 90 49 33
RVSP≤40
RVSP>40
Survival (%)
p<0.0001
Years of Follow Up
At risk
RVSP >40 23 11 8
RVSP ≤40 90 52 35
<Moderate LAVV Regurgitation
≥Moderate LAVV Regurgitation
Survival (%)
p=0.35
Years of Follow Up
At risk
≥Moderate LAVVR 40 20 13
<Moderate LAVVR 57 32 22
